# Long-term sensibility outcomes of secondary digital nerve reconstruction with sural nerve autografts: a retrospective study

**DOI:** 10.1007/s00068-021-01747-4

**Published:** 2021-07-19

**Authors:** Tomasz Dębski, Marcin Złotorowicz, Bartłomiej Henryk Noszczyk

**Affiliations:** 1 Department of Plastic Surgery, Orlowski Memorial Hospital, Medical Center of Postgraduate Education, Czerniakowska Street 231, Warsaw, 00-416 Poland; 2 Gruca Orthopaedic and Trauma Teaching Hospital, Medical Centre of Postgraduate Education, Otwock, Warsaw, Poland

**Keywords:** Secondary digital nerve reconstruction, Sural nerve autograft, Long-term surgical outcomes

## Abstract

**Background:**

Recovery of sensibility after digital nerve injury is crucial for restoring normal hand function. We evaluated long-term outcomes of digital nerve reconstruction with autografts.

**Methods:**

This retrospective study included patients who underwent secondary reconstruction of digital nerves with nerve autografting. Recovery of sensibility was evaluated based on the following: patient self-assessment, two-point discrimination (2PD), and a total sensation score (sum of proprioception, temperature sensation, and sharp/dull discrimination). Mixed models regression was used to study predictors of sensibility outcomes. The predictors analyzed were age, sex, smoking status, number of fingers involved in a patient (as a measure of injury severity), time to reconstruction, and time to follow-up.

**Results:**

In 61 patients, 174 digital nerves in 126 fingers were reconstructed after an average of 33.1 weeks from injury. The mean follow-up was 6.4 years from reconstruction. The mean graft length was 3.6 cm. Self-rated sensibility in the affected area was very good in 13% of patients, good in 33%, satisfactory in 40%, and poor in 24%. 2PD at 6 mm was present in 17% of patients, at 10 mm in 12%, and at 15 mm in 18% (mean 2PD was 10.8). Proprioception was preserved in 107 (85%) fingers, sensation of temperature was preserved in 99 (75%) of fingers, and sharp/dull discrimination in 88 (70%) fingers. Time from injury to reconstruction was the only significant predictor of the total sensation score.

**Conclusion:**

Our data indicate that earlier reconstruction is associated with a favorable outcome.

## Introduction

Traumatic hand injury is common, and it requires nerve reconstruction in about 6.1–10% of patients. The common and proper digital nerves are most frequently affected [1, 2].

Restoring normal sensibility is important for the recovery of hand function. Digital nerves commonly need to be surgically reconstructed, and intensive rehabilitation usually follows [1]. End-to-end coaptation and nerve grafting are the preferred methods of repairing severed nerves [1]. This method is recommended when the wound is clean, and both nerve ends are clearly visible and easily mobilized [3]. Where gaps exist, direct end-to-end coaptation cannot be used, and autografts or artificial nerve conduits are required for reconstruction. Nerve autografting is the gold standard for treating this type of injury, often with the sural nerve used as the graft when multiple nerves require repair [1, 3]. The implanted donor nerve, however, degenerates and cannot directly replace the severed nerve part [4]. Instead, it creates a supportive structure for the growing axons of the injured nerve, providing a growth-permitting scaffold, including Schwann cell basal laminae, neurotrophic factors, and adhesion molecules [4]. It is, therefore, unsurprising that sensory recovery following nerve repair is generally prolonged. A review of 14 studies of digital nerve repair showed that longer follow-up after trauma was a predictor of better functional recovery [3]. Excellent sensory recovery was only seen at follow-ups longer than 6 months [3].

In the current study, we reviewed medical records of patients who had undergone secondary digital nerve reconstruction. Our aim was to assess very long-term sensory outcomes of nerve autografting and to investigate selected patient and injury characteristics that may affect these outcomes.

## Methods

### Patients

**Fig. 1:**
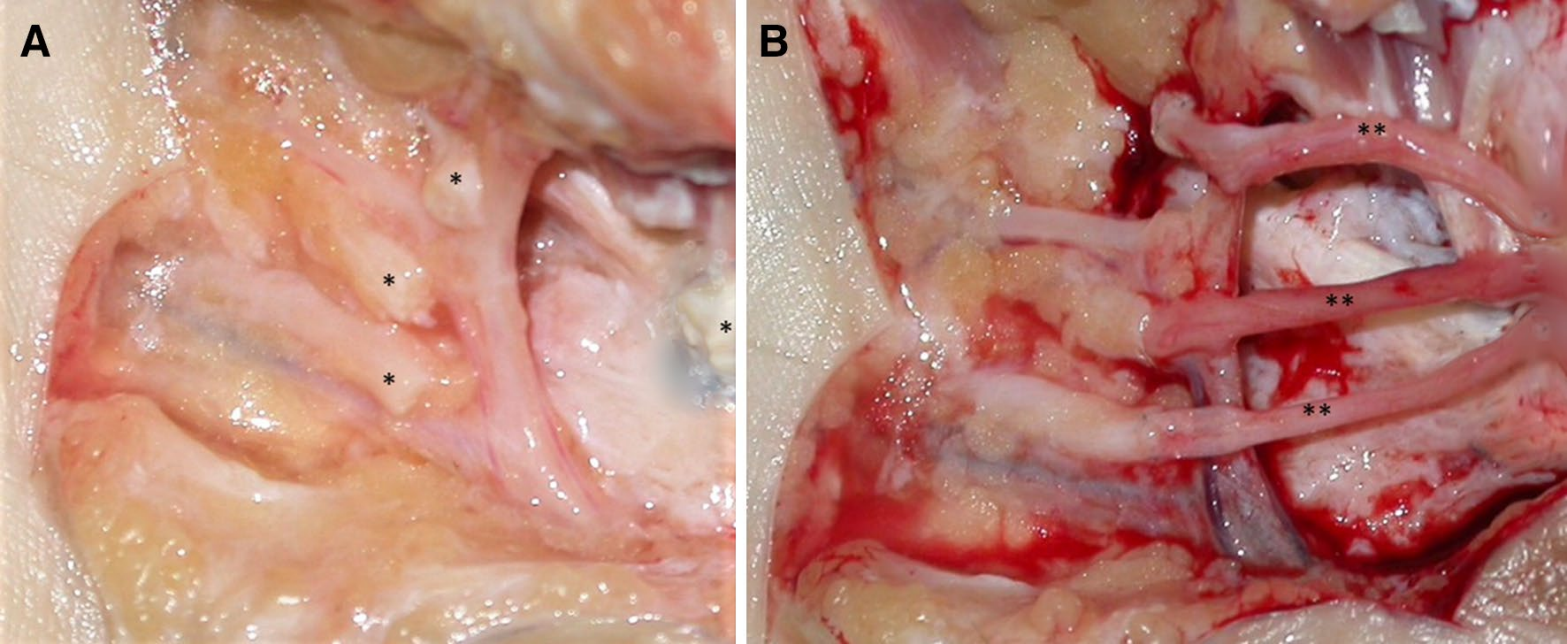
Common digital nerve reconstruction with sural nerve. **A** A gap between proximal and distal III, III, IV common digital nerve stumps (*) after refreshing, **B** nerve reconstruction with sural nerve autograft (**)

In this retrospective study, we sent invitation letters for a follow-up visit to all patients who had undergone secondary reconstruction of digital nerves with sural nerve autografting in our Department between 1994 and 2002. Primary nerve repair was not possible in these patients because of gap formation at the nerve injury site (mainly crash injury type) or unavailability of nerve grafting at the primary hospital. In all selected patients, contralateral sural nerves were used for reconstructions. Surgery was performed under regional anesthesia in a bloodless field (pneumatic cuff tourniquet) using surgical microscope (Leica, Austria). All injured nerves were dissected, both stumps were refreshed and epineural tensionless coaptation with 10-0 Prolene suture (Ethicon, USA) was performed (Fig. 1). After surgery, the hand was immobilized for 6 weeks in a dorsal blocking splint with a hand in a neutral position regardless of the site of injury. After immobilization patients received 3 months of physiotherapy. All surgeries were performed at the Plastic Surgery Department in Warsaw by surgeons with at least 3 years of training in digital nerve repair. Our hospital was, at the time of the study, a tertiary referral hospital providing services in the Mazovian district with a population of 5,356,838. As a referral hospital, we also admitted severe hand injury cases or cases after primary treatment from other districts in Poland [2]. Secondary digital nerve reconstruction with sural nerve was performed on an average of 10–15 patients per year. In cases primarily treated in our department stabilization of bone fractures, flexor/extensor tendons repair or soft tissue coverage reconstruction was performed first. Injured digital nerves were reconstructed after 1–3 months depending on the complexity of the case.

The study protocol was approved by the Bioethical Committee of the Medical Centre of Postgraduate Education in Warsaw and conducted in accordance with the Helsinki Declaration (No. 13/PB/2012).

### Assessments

We reviewed medical records of all patients included to obtain the following variables: age at reconstruction, sex, smoking status at reconstruction, graft length, primary injury mechanism, concomitant injuries to other hand structures, time from injury to reconstructive surgery, and time from surgery to follow-up. Before nerve reconstruction, all patients had absent sensibility in the area of reconstructed nerve.

During the follow-up visit, a standardized hand examination was performed as previously described [5–7]. The affected hands were examined for skin lesions, scars, fingernail abnormalities, and hypohydrosis. Patients completed a short questionnaire to assess sensibility within the fingers innervated by the reconstructed nerve as very good, good, satisfactory, or poor.

Two point discrimination (2PD) was assessed in each reconstructed nerve with a Dellon discriminator. Depending on which nerve was affected, the ulnar or radial side of the finger was touched with either one or both tips of instrument. The 2PD was scored as 1 (at 6 mm), 2 (at 10 mm), 3 (at 15), and 4 (> 15 mm).

Finally, in each reconstructed nerve, we tested three aspects of sensation: proprioception, temperature sensation, and sharp/dull discrimination. Proprioception was tested by asking the patient to close their eyes and tell their finger position during passive flexion or extension. The ability to sense temperature was tested by touching the skin area innervated by the reconstructed nerve with cotton wool swabs dampened with either warm (35 °C) or cold water (10 °C). The ability to discriminate between sharp and dull was tested in a pinprick test: we touched the skin innervated by the reconstructed nerve with either the tip or the head of a sharp pin and asked the patient to tell, with their eyes closed, which end of the pin was felt (sharp or dull). All aspects of sensation were rated as either present or absent, and were added to obtain a total sensation (TS) score (0, no sensation; 3, all aspects of sensation preserved).

### Statistical analysis

Descriptive statistics were calculated for all following variables: means (ranges), for continuous variables, and counts (percentages), for categorical variables. Mixed models regression was applied to study predictors of sensation after nerve reconstruction. We used data for individual nerves, with the subject as a random effect. The mean 2PD score and the TS score were used as dependent variables. The predictors (fixed effects) were age at reconstruction, sex, smoking status, number of fingers involved in a patient (as a measure of injury severity), time to reconstruction, and time to follow-up. *P* < 0.05 was considered statistically significant. Statistical analyses were performed with the R software (version 3.53).

## Results

### Patient characteristics

**Fig. 2:**
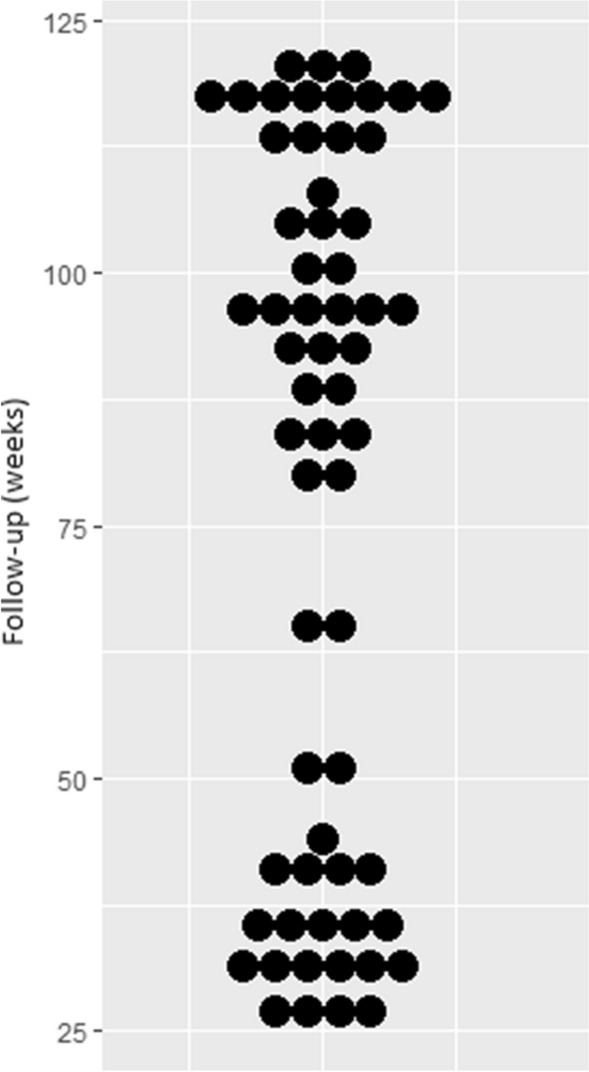
Scatter plot presenting spread of follow-up times

Of 86 patients invited, 61 (71%) reported for a follow-up visit. In total, they underwent 174 nerve reconstructions in 126 fingers. The mean time of follow-up was 6.4 years (2.2–10.1 years) (Fig. 2). Most patients were males (90.2%), and the mean age at reconstruction was 33.8 years. About half of the patients were smokers (52%). The mean time from injury to reconstruction was 33.1 weeks (5–430 weeks). Primary treatment before reconstruction was performed in our department in 22 patients (36%). Other 39 patients (64%) were referred from orthopedic (77%) or neurologic departments (23%) for secondary reconstruction. Among 174 repaired nerves, most were damaged in crush injuries (83%) or by cuts (16%). Additional hand structures were affected in nearly all patients (97%). The proper palmar digital nerves were injured more frequently than the common digital nerves (64% vs. 25%). The mean length of sural nerve grafts was 3.6 cm, although grafts up to 9 cm in length were required in some patients. The characteristics of patients at nerve reconstruction are shown in Table 1.

**Table 1:** Baseline patient and injury characteristics

Patient characteristics	Total *n* = 61
Male sex, *n* (%)	55 (90%)
Age at reconstruction, mean	33.8 years (17–63 years)
Smoker, *n* (%)	32 (52%)
Time from injury to reconstruction, mean	33.1 weeks (5–430 weeks)
Time from reconstruction to follow-up assessment, mean	6.4 years (2.2–10.1 years)
Dominant hand injury, *n* (%)	31 (51%)
Palmar digital nerves damaged, *n* (%)	
Common	15 (25%)
Proper	39 (64%)
Both	7 (11%)
Number of nerves reconstructed per patient, *n* (%)	
1	17 (28%)
2	13 (21%)
3	13 (21%)
4	8 (13%)
≥ 5	10 (16%)
Graft length, mean (range)	3.6 cm (1–9 cm)
Isolated nerve injury, *n* (%)	6 (3%)
Primary injury type, *n* (%)	
Crush	145 (83%)
Cut	28 (16%)
Gunshot	1 (< 1%)
Nerve reconstruction following digital replantation, *n* (%)	10 (6%)
Nerve reconstruction following digital revascularisation, *n* (%)	9 (5%)

Self-assessed sensibility in the digits innervated by the reconstructed nerves was very good in 8 (13%) patients, good in 20 (33%), satisfactory in 18 (30%), and poor in 39 (24%).

### Assessment of hand and donor site function

Physical examination of the digits innervated by the reconstructed nerves revealed constricting scars in 15 (25%) of patients. The skin of the affected fingers was completely dry in 3 (5%) patients, drier than in the unaffected digits in 15 (25%), and similar as in the unaffected fingers in 43 (70%). Complaints related to the donor site were reported by 9 (15%) of patients, and they included tingling or sensory deficit in the skin area innervated by the sural nerve.

Among the 174 reconstructed nerves, the 2PD score was 1 in 30 (17%) nerves, 2 in 21 (12%) nerves, 3 in 31 (18%) nerves, 4 in 92 (53%) nerves. Among nerves with preserved 2PD (*n* = 82), the mean 2PD was 10.8 mm. Proprioception was preserved in 148 (85%) nerves, sensation of temperature was preserved in 130 (75%) nerves, and sharp/dull discrimination in 122 (70%) nerves. The mean (± standard deviation) TS score was 2.35 ± 1.00.

### Predictors of sensation after nerve reconstruction

Longer time from injury to reconstruction was a significant predictor of decreased TS scores, but not of 2PD scores at follow-up (Table 2). None of the remaining variables was significantly associated with the 2PD or TS scores, although the number of fingers injured was related to lower TS scores at the border of statistical significance (*P* = 0.054; Tables 2, 3).

**Table 2:** Predictors of the TS score (proprioception, temperature, sharp/dull discrimination) at follow-up. Statistical significance (*p*<0.05) are bolded

Predictor (fixed factor)	Estimate	Standard error	Degrees of freedom	*T* value	*P*
Number of fingers injured	− 0.236	0.119	49.650	− 1.971	0.054
Smoking	0.094	0.234	49.504	0.401	0.689
Age at reconstruction	0.006	0.009	50.210	0.669	0.506
Graft length per nerve (cm)	0.002	0.035	84.450	0.061	0.951
Time to reconstruction (weeks)	− **0.005**	**0.002**	**55.818**	− **2.372**	**0.021**
Time to follow-up (weeks)	− 0.003	0.003	50.314	− 0.812	0.420

**Table 3:** Predictors of 2-point discrimination at follow-up

Predictor (fixed factor)	Estimate	Standard error	Degrees of freedom	*T* value	*P*
Number of fingers injured	0.106	0.157	42.759	0.675	0.503
Smoking	− 0.276	0.300	43.020	− 0.919	0.363
Age at reconstruction	− 0.001	0.012	45.373	− 0.117	0.907
Graft length per nerve (cm)	0.002	0.048	60.679	0.036	0.971
Time to reconstruction (weeks)	0.003	0.002	58.593	1.275	0.207
Time to follow-up (weeks)	0.002	0.004	44.605	0.644	0.522

## Discussion

In this study, we found that shorter times from injury to surgery were associated with better sensation in the long-term among patients who underwent secondary digital nerve reconstruction. This association was significant for a TS score that included proprioception, temperature sensation, and sharp-dull discrimination, but it was not significant for 2PD.

Although primary repair of peripheral nerves offers improved clinical outcomes compared to secondary reconstruction [8], it is not always feasible in patients presenting with large, contaminated, or crush wounds or when the neighboring hand structures are substantially affected. In these patients, secondary repair may be indicated. Our study reports very long-term (up to 10 years) outcomes of secondary digital nerve reconstruction with nerve autografts. Although, in our study, protective sensation recovered in nearly all patients, 2PD was restored only in about half of them, which suggests incomplete sensory recovery. Although 2PD is widely used to test sensory function, other measures, like the modified Highet classification [9], should perhaps be used in addition to 2PD [1]. Such measures take into account the ability to sense touch, pain or temperature, or hyperalgesia [1, 9].

Paprottka et al. performed a systematic review and meta-analysis to compare outcomes of digital nerve reconstruction after different procedures, including nerve grafting [1]. In that meta-analysis, the percentage of patients with good to excellent sensory recovery ranged between 14 and 67% [1]. More recently, Stang et al. reported that, of 28 patients who received a posterior interosseous nerve graft or medial antebrachial cutaneous nerve graft, 46% achieved S4 sensibility and 36% achieved S3 + sensibility, on the modified Highet classification [10]. In our study, where a 2PD result < 15 mm was achieved, the modified Highet score would be S3 + (30%, reconstruction cases with good and satisfactory 2PD result), or S4 (17%, very good 2PD result). Thus, our results are similar to those of other studies on nerve grafting for digital nerve repair. The mean 2PD of 10.8 mm, in our study, was also similar to previous reports. After a mean follow-up of 23 months, Chen et al. reported a mean 2PD score of 9.2 mm in 31 patients with 38 proper digital nerve defects, who underwent sural nerve grafting [11]. Another study by the same authors reported a mean 2PD score of 9.4 mm in 27 patients with injuries to proper digital nerves and the thumb who received sural or medial antebrachial cutaneous nerve grafts [12].

Fifteen percent of patients in our study reported problems with the donor site. Although there are sources of shorter nerve grafts in the upper extremity (cutaneous antebrachial nerve or dorsal interosseous nerve), which reduce donor site morbidity, we consider the sural nerve a useful source of long grafts required for the repair of injuries affecting multiple nerves or those associated with substantial gaps between severed nerve ends (in our study mean length of sural nerve grafts was 3.6 cm). Donor site morbidity in our study compares favorably with two earlier reports, in which, after several years from sural nerve harvesting, 5–17% of patients reported pain, 5–34% reported cold sensitivity, 24–48% reported discomfort, tingling, or increased skin sensation, and 17% reported minimal levels of functional impairment [13, 14].

A range of commercial products, including de-cellularized nerve allografts and collagen-based or synthetic nerve guides/conduits, have been developed, and their application in peripheral nerve repair has been extensively reviewed (see for example Arslantunali, et al. Khoe et al. and Kaushik and Hammert) [15–17]. Although autologous tissues, such as muscles and/or veins, may also be used to form a nerve conduit, commercial products are available off-the-shelf and minimize the time needed for tissue harvesting and conduit preparation. These products may substitute for nerve autografts, allowing the surgeon to bridge the gap between severed ends of a nerve without the need to denervate another area and to avoid donor site morbidity. Selected prospective and registry-based studies have reported high rates of meaningful recovery (S3 + or S4 on the modified Highet scale) with commercially available products for bridging nerve gaps: 84% with processed nerve allografts [18], 63% with collagen nerve conduits [19], and 74% with synthetic conduits [20]. However, despite a wealth of published literature, considerable differences between individual studies exist in terms of utilizing primary vs. secondary repair, average gap length and patient characteristics, which precludes a robust comparison of different repair methods. A large, prospective randomized study of different options for bridging digital nerve gaps, stratified by key prognostic factors, could provide information on the value of different techniques, including autografting.

In addition to describing long-term outcomes of nerve grafting, we also investigated patient- and injury-related factors that could affect sensory recovery. We found that the time from injury to reconstruction was significantly related to the total sensation (TS) score that included proprioception, temperature sensation, and sharp/dull discrimination. In contrast, we did not find any predictors of 2PD discrimination. Our findings suggest that 2PD and other types of sensation may be restored differentially by digital nerve reconstruction. The sensation of temperature, for example, is a protective sensation, which forms an earlier step towards full sensory recovery according to the modified Highet classification [9] than fine, discriminative touch examined with the 2PD test. Moreover, no other predictors of sensibility after nerve reconstruction in our study proved significant, including smoking. This observation contrasts with some previous studies [21–24]. Similarly, younger age at reconstruction [3, 24, 25], shorter grafts [25], and isolated nerve injury [24] have all been found to be associated with favorable sensory outcomes after nerve reconstruction within the upper limb. The follow-up time in our study was also not related to sensory outcomes, which is in line with the report by Bulut et al. [24], but does not agree with the observations reported by Meermans et al. [3]. Although graft length was not found to be significantly associated with sensory recovery in our study, previous studies reported an association between sensory recovery and the length of the gap between severed nerve ends. He et al. found shorter gap length to be an independent predictor of good to excellent sensory recovery after the repair of both purely sensory and mixed nerves [25]. The review by Mermans et al. identified a tendency (not statistically significant) for worse sensory recovery with increasing length of gap [3]. Female sex has also been associated with significantly better sensory recovery compared with males, likely due to lower injury severity in women, who usually perform only light manual labor, or due to women showing better compliance with postoperative treatment [25]. As women constituted only a tenth of the patients in our study, this sex imbalance could have affected our results. Finally, a recent study by Fakin et al., investigating sensory outcomes following end-to-end coaptation of 93 digital nerves [26], found surgeon experience to be the only significant predictor of sensory outcome when accounted for age, smoking, mechanism of injury, lesions or anastomosis of digital artery, or time of immobilization [26]. This finding highlights the importance of treating patients with digital nerve injuries at specialized centers with teams experienced in repairing small peripheral nerves. Our study was conducted at a specialist reconstructive surgery center, and all surgeons had at least 3 years of training in digital nerve repair.

Our study was limited because it was observational, retrospective, and single-center. Of 86 patients, only 61 (71%) reported for follow-up, which could lead to bias. Moreover, we did not evaluate surgical outcomes with regards to activities of daily live. Furthermore, the effect of surgeon experience on sensory outcome was not assessed. The strengths of our study include a relatively large sample size and a long follow up.

## Conclusions

This study presents long-term outcomes of secondary digital nerve repair with nerve autografts, which might be valuable to patients and clinicians. Although three quarters of patients rated reconstruction outcomes as at least satisfactory, 2PD was poor in approximately half of the patients. Only the time from injury to reconstruction was a significant predictor of sensibility (proprioception, temperature sensation, sharp/dull discrimination). We did not find any significant predictors of 2PD.
